# Study on the Effect of Nanocellulose Type and Dosage on the Stability Performance of Cemented Backfill with Lithium Tailings

**DOI:** 10.3390/ma19194256

**Published:** 2026-10-08

**Authors:** Cong Xiao, Qing Deng, Xuefangzi Dong, Shibin Zhang, Daopei Zhu

**Affiliations:** 1 School of Civil Engineering, Hubei Engineering University, Xiaogan 432000, China; 2 School of Civil Engineering and Surveying & Mapping Engineering (Nanchang), Jiangxi University of Science and Technology, Ganzhou 341000, China

**Keywords:** nanocellulose, uniaxial compressive strength, cemented tailings backfill (CTB), microstructure

## Abstract

The large-scale accumulation of lithium-feldspar tailings generated during lithium ore beneficiation poses environmental and resource-utilization challenges. Cemented lithium-feldspar tailings backfill (CLFTB) provides an effective approach for their underground disposal and resource utilization; however, its mechanical performance remains a key concern. Nanocellulose, with its high specific surface area and fibrillar structure, has potential for improving the stability and mechanical properties of cementitious tailings backfill. This study investigates the effects of nanocellulose type and dosage on the macro- and micro-scale performance of CLFTB. Three nanocellulose types, namely carboxylated cellulose nanofibrils (CNFs), cellulose nanocrystals (CNCs), and microfibrillated cellulose (MFC), were incorporated at dosages of 0.6–1.5 kg/m^3^. Uniaxial compressive-strength tests and microstructural analyses were conducted to evaluate their effects. The results showed that all nanocellulose-modified groups exhibited higher compressive strength than the control group. The optimum dosage was 0.9 kg/m^3^, while MFC provided the greatest strength enhancement among the three nanocellulose types. Nanocellulose incorporation also altered the failure behavior and mitigated crack propagation. EDS analysis further revealed differences in the local elemental characteristics of the modified specimens. Overall, the results demonstrate that nanocellulose, particularly MFC, can effectively enhance the mechanical performance and stability of CLFTB.

## 1. Introduction

With the accelerated global energy transition, lithium demand as a strategic critical mineral has exhibited exponential growth during the beneficiation and metallurgical processes of spodumene ores. Associated lithium-feldspar tailings (Li-feldspar tailings) generated as the primary industrial by-product has become a critical environmental challenge, impeding the industry’s sustainable development due to their storage and disposal requirements [1]. Statistical data indicate that lithium-feldspar tailings in China currently account for over 18% of the total industrial solid waste. The long-term stockpiling of these tailings has led to critical environmental governance challenges, including heavy-metal migration, dust pollution, and land occupation, which urgently require resolution [2]. To address the resource utilization of lithium tailings, the academic community has explored multiple pathways: the first focuses on transformation into construction materials through phase reconstruction to achieve value-added utilization of tailings in the building materials sector; the second involves the application of backfill mining technology, emphasizing the optimization of engineering performance in cemented-tailings backfill (CTB) [3]. Among these, CTB technology has garnered significant attention due to its dual environmental and economic benefits. CTB is a technique employed in underground mining that involves scientifically proportioning tailings, binder materials, and mixing water to form an underground backfill structure capable of providing both structural support and enabling resource recovery [4]. Cemented Lithium-Feldspar Tailings Backfill (CLFTB) is a composite matrix formed by mixing cementitious materials (e.g., cement, fly ash), spodumene tailings, and water. While retaining the conventional characteristics of CTB, CLFTB specifically targets the recycling of lithium-processing tailings. As a specialized form of CTB, established research methodologies for CTB specimens remain fully applicable to CLFTB. The technical advantages of CTB are demonstrated in: (1) achieving in situ disposal of over 50% of mineral processing tailings; (2) recovering residual precious metals through metal leaching processes [5]; and (3) reducing cement consumption by 30–40% compared to traditional mining methods [6].

CTB significantly mitigates the negative environmental impacts of mining activities through the resource utilization of tailings waste [7]. The network structure formed by its cementitious hydration reaction provides early bearing capacity [8]. Under certain conditions, customized design of Cemented Tailings Backfill (CTB) can be achieved by varying the type of tailings and the curing period [9]. Upon hardening, CTB enhances surrounding rock stability via internal stress redistribution [10]. The optimization of CTB’s mechanical properties, serving as its technical core, is primarily governed by three key factors: (1) Material intrinsic parameters: including tailings particle size distribution (D_10_–D_90_ range), binder type, and binder-to-tailings ratio (1:4–1:10) [6,11]. (2) Process conditions: such as slurry concentration, curing temperature/humidity, and placement density [12,13]. (3) Environmental coupling effects: the long-term hydro–mechanical–chemical (HMC) coupling interactions during service significantly influence its durability [14].

### 1.1. Nanocellulose-Based Reinforcement of Cementitious Materials

However, CTB still faces critical technical challenges, particularly its relatively low tensile resistance and brittle failure behavior under compressive loading [15]. Nanocellulose materials (NCMs), owing to their nanoscale dimensions, high specific surface area, abundant surface hydroxyl groups, and fibrillar structure, have recently attracted increasing attention as reinforcement additives for cement-based materials [16,17,18,19]. Unlike conventional fibers, nanocellulose can interact with the cementitious matrix at the micro- and nanoscale, potentially promoting the formation of a more continuous matrix, improving interfacial bonding, refining pores, and restricting microcrack propagation. Previous studies have demonstrated that nanocellulose can improve the mechanical performance of cementitious materials through a combination of physical bridging, matrix densification, and modification of hydration processes [20,21].

In addition to their potential mechanical reinforcement effects, cellulose-based materials may provide additional functional benefits in cementitious composites, particularly in relation to thermal properties. Previous studies on bio-based additives and cellulose-containing cementitious materials have reported changes in thermal conductivity associated with the incorporation of biomass-derived materials and the resulting modification of the internal microstructure. For example, biochar derived from Washingtonia plant biomass waste has been investigated as a reinforcement in bio-mortar, demonstrating the potential of biomass-derived materials to simultaneously modify mechanical and thermal properties [22]. These effects are generally related to the intrinsic thermal characteristics of the bio-based phase as well as changes in porosity, density, moisture distribution, and heat-transfer pathways within the cementitious matrix. Therefore, the incorporation of cellulose-based materials may offer potential thermal-regulation or insulation benefits in addition to mechanical reinforcement. However, the thermal behavior of nanocellulose-modified cemented-tailings backfill remains insufficiently understood and is influenced by factors such as nanocellulose type and dosage, moisture content, porosity, and matrix composition. Since the present study focuses on the mechanical performance and failure behavior of CLFTB, thermal conductivity was not experimentally measured and is therefore not quantitatively evaluated herein.

### 1.2. Characteristics of Different Nanocellulose Materials

Importantly, nanocellulose is not a single material but a family of cellulose-based materials with substantially different morphologies, dimensions, and surface characteristics. In this study, three representative types were selected: carboxylated cellulose nanofibrils (CNFs), cellulose nanocrystals (CNCs), and microfibrillated cellulose (MFC). CNF consists of long, flexible nanofibrils with diameters of approximately 3–20 nm and lengths exceeding 1 μm; its carboxyl groups can improve dispersion in aqueous systems and its high aspect ratio favors the formation of an interconnected fibrillar network. CNC has a similar nanoscale diameter but a much shorter length of approximately 100–300 nm and predominantly hydroxyl-functionalized surfaces, giving it a relatively rigid, crystalline morphology. In contrast, MFC has a substantially larger fibril diameter (0.1–1 μm) and length (>20 μm), providing a longer and more extended fibrous structure [23,24,25,26,27,28,29,30,31,32,33,34,35]. These differences in aspect ratio, rigidity, surface functionality, and network-forming ability may lead to different interactions with cement hydration products and different abilities to bridge defects and modify the pore structure. Therefore, directly comparing CNF, CNC, and MFC under the same CLFTB conditions is necessary to clarify whether the type-dependent characteristics of nanocellulose translate into differences in strength enhancement and failure behavior.

### 1.3. Research Gap and Objectives

Although previous research has confirmed the reinforcing potential of nanocellulose in cementitious materials, the type-dependent effects of different nanocellulose materials in cemented lithium-feldspar tailings backfill (CLFTB) remain insufficiently understood. In particular, lithium-feldspar tailings have a distinct particle-size distribution and mineralogical composition, which may influence the dispersion, interfacial interaction, and reinforcing efficiency of nanocellulose within the cemented matrix. Existing studies have mainly focused on conventional cementitious systems or have investigated nanocellulose reinforcement in general cemented tailings backfill [36], while research on lithium feldspar tailings-based backfill has more commonly considered conventional fibers and their influence on early-age mechanical performance [37]. Consequently, the relative effectiveness of CNF, CNC, and MFC in CLFTB, as well as the influence of their dosage on strength development and failure behavior, has not yet been systematically established.

Therefore, this study employed uniaxial compressive strength (UCS) testing on three distinct nanocellulose-reinforced CLFTB samples to evaluate their mechanical properties. The experiment employed four NCM concentrations (0.6, 0.9, 1.2, and 1.5 kg/m^3^), with samples cured for 7 days before testing. Evaluating the 7-day early-age strength of CLFTB provides a reliable predictive basis for assessing its strength-development potential and long-term durability performance [38]. This approach aids in optimizing construction scheduling and supports the selection of appropriate curing techniques, thereby highlighting the critical importance of early-age strength performance research in backfill engineering [39].

Based on the experimental results, this study analyzes the influence of different nanocellulose types and contents on the stress–strain curves and peak strain of CLFTB specimens and examines the damage behavior of fiber-reinforced CLFTB samples. Finally, the microstructure of nanocellulose-reinforced CLFTB was observed using Scanning Electron Microscopy (SEM). Given that curing duration significantly affects compressive strength, all specimens in this experiment underwent a standardized curing period of 7 days. Targeting the resource utilization of lithium tailings, this research employs an innovative nanocellulose reinforcement technology to markedly improve the mechanical performance of cement-based backfill. A particular focus on early-age strength development yielded patterns that serve as a reliable quantitative basis for evaluating long-term strength potential and forecasting structural durability. These findings establish a vital theoretical groundwork for implementing low-cost, high-performance tailings backfill in engineering practice.

## 2. Experimental Program

### 2.1. Material Properties

The raw materials used for preparing CLFTB specimens in this experiment include lithium-feldspar tailings (LFTs), nanocellulose, and ordinary Portland cement conforming to the 42.5 R standard—where “R” denotes rapid early-strength development—along with water as an essential component. The production process utilized instruments such as an electronic balance, beakers, a cement mortar mixer, casting molds, and a curing chamber.

**Figure 1:**
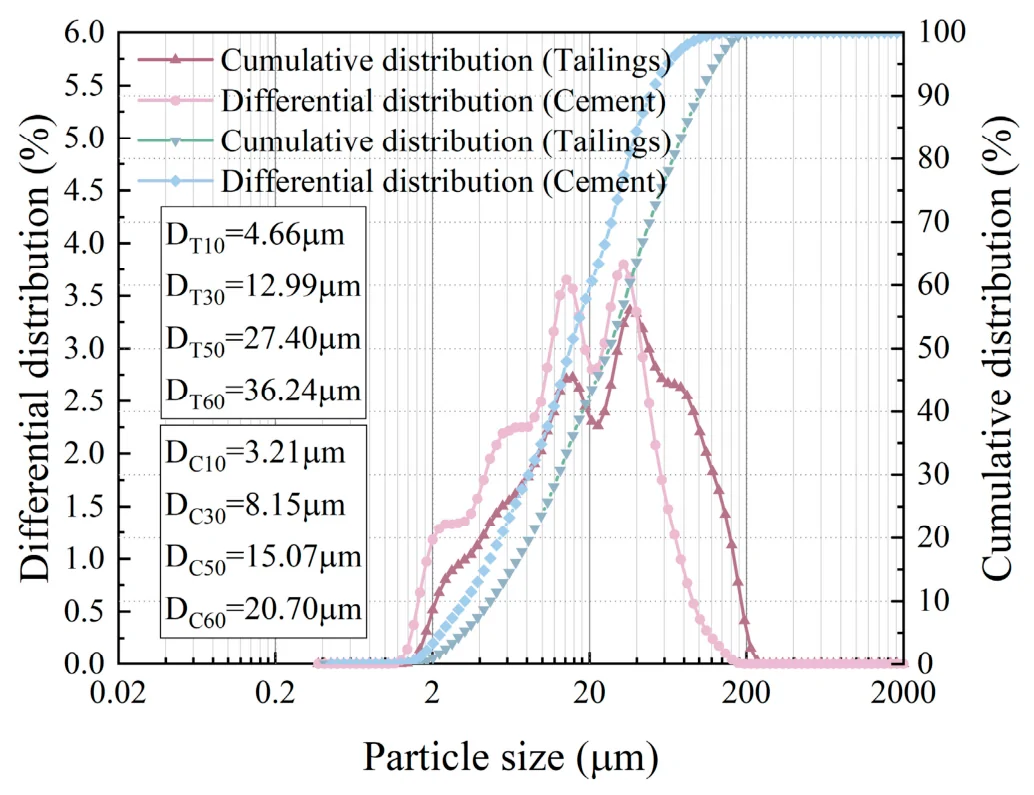
Particle size distribution of LFT.

The lithium-feldspar tailings used in this study were collected from an industrial mining site in Yichun, Jiangxi Province, China, and represent the residual material obtained after the beneficiation and lithium-recovery processes rather than the original lithium-bearing ore. During beneficiation, lithium-bearing minerals are preferentially separated and recovered, resulting in a relatively low Li_2_O content (0.26%) in the residual tailings used for backfilling. Particle size analysis via a laser-diffraction method revealed the distribution curve shown in Figure 1. Subscripts *T* and *C* denote the tailings (T) and cement (C) systems, respectively, with D T10 and D C10 representing the critical particle sizes at 10% cumulative sieve residue. Analysis of particle-size characteristic parameters indicates that the curvature coefficient of the tailings system closely aligns with that of cement, but its non-uniformity coefficient is significantly higher, reflecting greater discreteness in tailings gradation. Both systems exhibit typical multi-modal distribution characteristics, likely attributable to differences in mineral composition and grinding process parameters. The chemical composition of the lithium feldspar tailings was determined using X-ray fluorescence (XRF) spectroscopy. Before testing, the samples were dried to constant mass and ground into a fine powder to ensure sample homogeneity. The prepared powder was then subjected to XRF analysis to determine the contents of the major oxides, including SiO_2_, Al_2_O_3_, CaO, Fe_2_O_3_, MgO, SO_3_, and Li_2_O. The loss on ignition (LOI) was determined separately by heating the dried sample at high temperature, and the mass loss was calculated as the percentage difference between the initial mass and the mass after ignition. The chemical composition and LOI of the lithium feldspar tailings are summarized in Table 1. It should be noted that the chemical composition of lithium tailings can vary considerably depending on the ore mineralogy, beneficiation process, and lithium-recovery efficiency.

Nanocellulose materials: This experiment utilized three types of NCM—carboxylated cellulose nanofibrils (CNFs), cellulose nanocrystals (CNCs), and microfibrillated cellulose (MFC). The specific performance indicators of these nanocellulose materials are listed in Table 2.

### 2.2. Sample Preparation

The experiment employed P.O 42.5 ordinary Portland cement and lithium tailings as matrix materials. The binder-to-tailings ratio was set at 1:6 by mass. Although Wang et al. [36] adopted a binder-to-tailings ratio of 1:8, a relatively higher binder content was used in the present study to provide sufficient early-age strength for the lithium-feldspar tailings-based backfill system. The binder-to-tailings ratio was kept constant for all mixtures to isolate the effects of nanocellulose type and dosage. Based on the dosage levels investigated by Wang et al. [36], four nanocellulose dosages of 0.6, 0.9, 1.2, and 1.5 kg/m^3^ were selected in the present study. These four levels cover a relatively low-to-high dosage range and allow the dosage-dependent effects of nanocellulose to be systematically evaluated. The same dosage levels were applied to CNF, CNC, and MFC to facilitate a direct comparison of their reinforcing effects under identical mixture conditions. Polypropylene demountable cylindrical molds (dimensions: 50 mm diameter × 100 mm height, compliant with ASTM D4832 standards) were uniformly coated with a silicone-based release agent on their inner walls 24 h before casting to minimize demolding interface damage. Cement (100.00 g), tailings (600.00 g), nanocellulose, and water (233.33 g, 75% mass concentration) were precisely weighed using an electronic balance with 0.0001 g accuracy. To prevent uneven nanocellulose distribution due to density differences, cement, tailings, and nanocellulose were dry-mixed in a mixer for 3 min to ensure uniform fiber dispersion within the aggregate system. Water was then gradually added to the dry mixture, followed by three additional minutes of mixing. The slurry was poured into molds in three layers, each compacted for 30 s using a vibrating table (frequency: 50 Hz, amplitude: 2 mm) to eliminate air bubbles. Molds were placed in a curing chamber under constant temperature (20 ± 3 °C) and humidity (90 ± 3%). After 48 h, backfill specimens were de-molded and stored in the curing chamber for 7 days. Detailed specimen masses are provided in Table 3.

**Figure 2:**
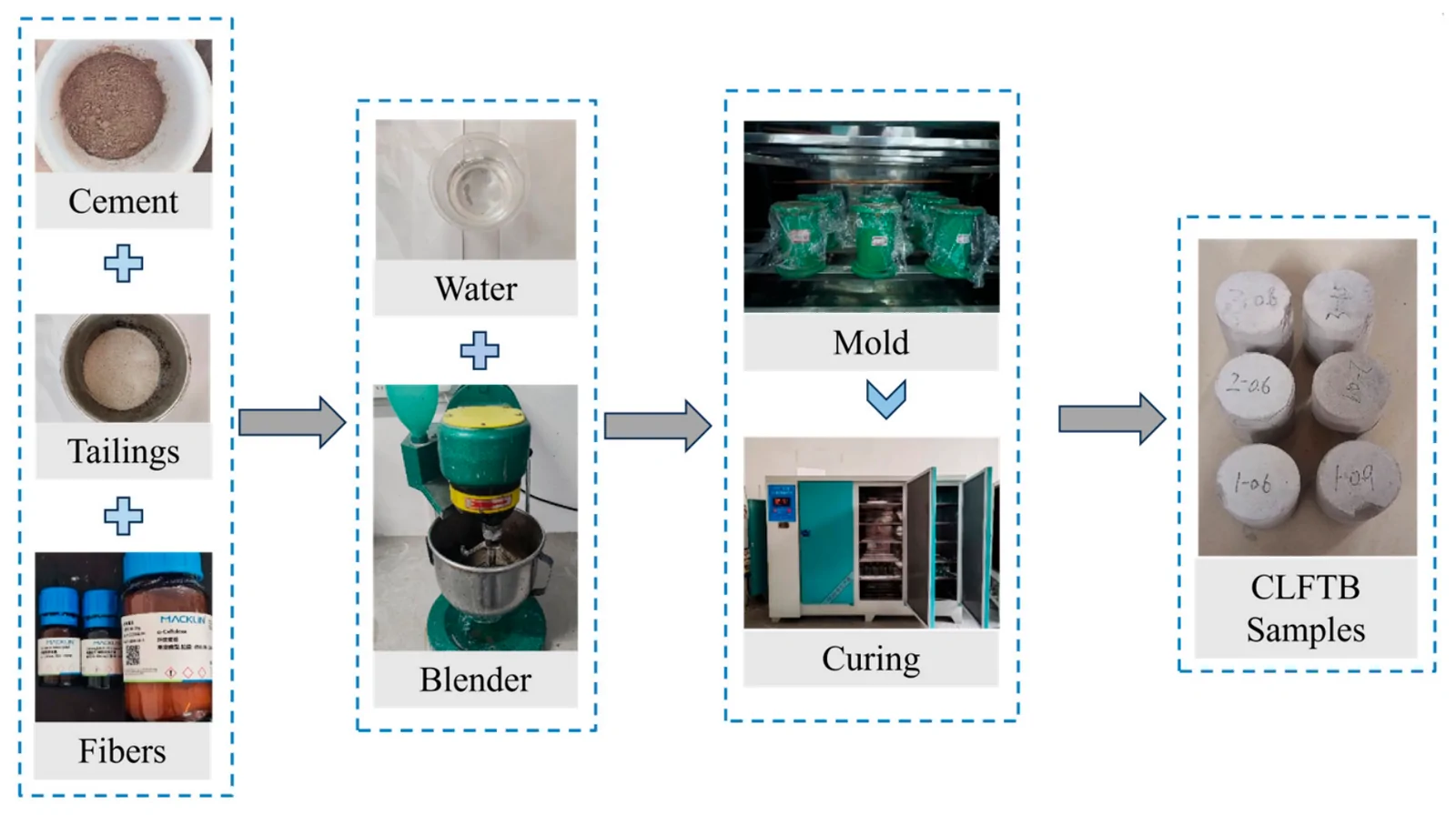
Material composition and specimen-preparation protocols.

In accordance with Article 5.3.2 of the National Standard GB/T 51450-2022: Technical Code for Backfill Engineering in Metal and Nonmetal Mines, the slurry casting process strictly controlled fluidity (spread diameter: 550 ± 20 mm) to prevent overflow. Each casting layer height did not exceed two-thirds of the mold height, with layered vibration (30 s per layer) applied to eliminate internal air gaps. Immediately after molding, specimens were fully wrapped with a 0.05-mm thick polyethylene film to prevent moisture evaporation-induced plastic-shrinkage cracks. Upon curing completion, samples were extracted and ground on both ends using a double-face grinding machine to ensure parallelism error ≤ 0.05 mm (compliant with ISO 4012 specifications), thereby eliminating contact stress concentration effects on UCS testing. The experiment required 39 specimens in total, including three cellulose-free controls for baseline performance comparison. Specimens were divided into four groups based on nanocellulose dosages (0.6, 0.9, 1.2, and 1.5 kg/m^3^), with three replicates per group. Notably, if any specimen’s UCS value deviated by ±15% from the group mean, additional specimens were prepared. Cylindrical specimens with a diameter of 50 mm and a height of 100 mm were prepared for the UCS tests. The specimen preparation process, including mold preparation, slurry pouring, vibration, demolding, and curing, is illustrated in Figure 2.

### 2.3. Research Methodologies

#### 2.3.1. UCS Experiment

Unconfined Compressive Strength (UCS) testing was conducted in compliance with ASTM D2166 standards using a GCTS STX-1000 electronic universal testing machine. This test quantifies the peak compressive stress of specimens under unconfined conditions to characterize the macroscopic mechanical response of backfill materials under quasi-static loading. As illustrated in the setup, axial load was applied at a constant rate of 0.5 mm/min in displacement-controlled mode via the computer-controlled testing system. Real-time stress–strain curves were generated by the control software, with the peak stress during specimen failure recorded as the UCS value.

#### 2.3.2. SEM-EDS Analysis

To investigate the mechanism of NCM on the microstructure of CLFTB, this study employed a Zeiss GeminiSEM 360 field-emission scanning electron microscope (FE-SEM) for microstructural morphology and elemental distribution characterization. During sample pretreatment, a five-nm-thick Au–Pd alloy coating was deposited under a vacuum using an ion sputter coater (Leica EM ACE600) to mitigate charging effects and enhance secondary electron signal intensity. Imaging was performed in secondary electron mode (SE2) with parameters set to an accelerating voltage of 15.00 kV, beam current of 1.0 nA, working distance of 11.0 mm, and magnification of 5000× to capture real-time microstructural features of the pore–fiber interfacial regions. Simultaneously, energy-dispersive X-ray spectroscopy (EDS) area-scanning mode was activated to generate 2D elemental distribution maps of C, O, Si, and Ca, enabling analysis of interfacial bonding between cellulose and cement hydration products. All specimen observation surfaces underwent vacuum impregnation with epoxy resin followed by polishing to ensure cross-sectional flatness errors below 0.1 μm (per ASTM E3-11 standards).

## 3. Results and Discussion

### 3.1. Mechanical Performance of NCM-Stabilized Backfills Under Uniaxial Compression

**Figure 3:**
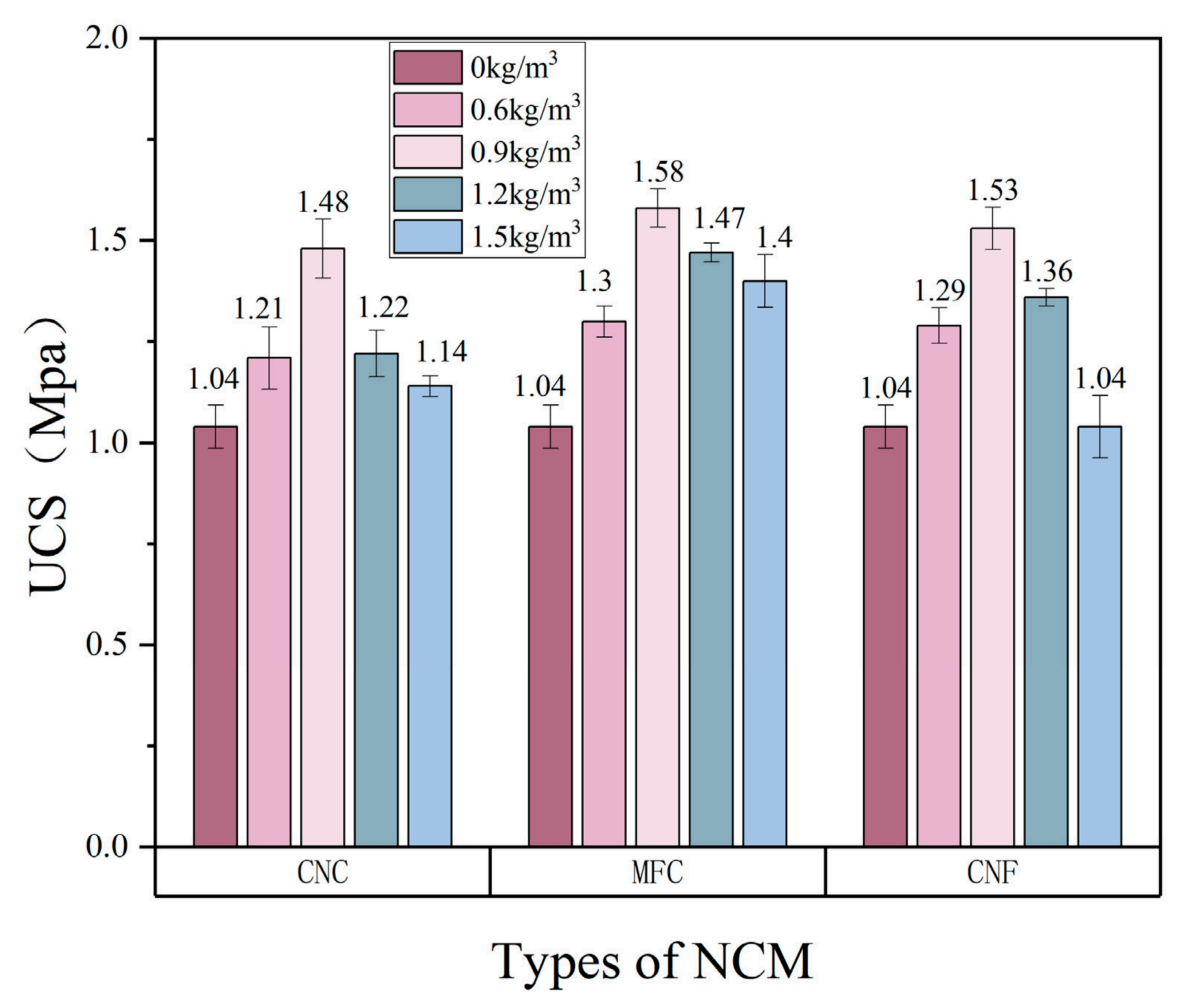
Effect of CNC, MFC, and CNF on the UCS of CLFTBs.

Based on the uniaxial compressive strength (UCS) test results of CLFTB presented in Figure 3, this study systematically elucidates the regulatory effects of NCM type and concentration on the mechanical properties of CLFTB. The bar chart displays NCM types (CNC, MFC, CNF) along the horizontal axis, concentration gradients (0–1.5 kg/m^3^) through color coding, and quantifies compressive strength values on the vertical axis. Control specimens without NCM exhibited a compressive strength of 1.040 MPa, consistently lower than NCM-modified samples. All three nanocellulose types demonstrated broadly similar enhancement trends for CLFTB’s UCS performance. At 0.6 kg/m^3^ concentration, significant strengthening effects were observed, with UCS values reaching 1.21 MPa (CNC), 1.30 MPa (MFC), and 1.29 MPa (CNF), representing 16.3%–25.0% improvements over the control group. Peak strengths occurred at 0.9 kg/m^3^: CNC (1.48 MPa), MFC (1.58 MPa), and CNF (1.53 MPa). Further increasing concentration to 1.5 kg/m^3^ induced concentration-dependent strength attenuation, with CNF specimens showing the most pronounced degradation. Notably, MFC specimens maintained structurally applicable strength at 1.40 MPa, meeting the ≥1.0 MPa minimum strength requirement specified in Section 5.2.3 of China’s Technical Code for Cemented Total Tailings Backfill (SAC/TC 379-2020).

At an NCM concentration of 1.2 kg/m^3^, the compressive strengths of the three CLFTB specimens reached 1.22 MPa (CNC), 1.47 MPa (MFC), and 1.36 MPa (CNF), marking the onset of strength reduction across all formulations. When concentration increased to 1.5 kg/m^3^, compressive strengths further declined to their minimum within the experimental range: 1.14 MPa (CNC), 1.40 MPa (MFC), and 1.04 MPa (CNF). Notably, all NCM types and concentrations within the tested range enhanced the mechanical properties of CLFTB compared to the unmodified control. Peak performance occurred universally at 0.9 kg/m^3^ NCM concentration. Regarding material efficacy, MFC demonstrated the strongest UCS enhancement at this optimal concentration, consistent with existing literature [40], while CNC exhibited the weakest improvement. Crucially, all cellulose-reinforced CLFTB specimens satisfied the engineering requirement of ≥1.0 MPa compressive strength as stipulated by the National Gold Standardization Technical Committee (SAC/TC 379, 2020). Experimental reliability was ensured through concurrent testing under identical environmental conditions, with reported data representing the mean values of triplicate specimens, thereby minimizing operator-induced variability.

The two-way ANOVA results indicate that NCM type, NCM dosage, and their interaction significantly affected the UCS of the cemented lithium-tailings backfill (*p* < 0.001), as shown in Table 4. Among the investigated factors, NCM dosage produced the largest contribution to the variation in UCS, followed by NCM type, while the significant interaction effect indicates that the influence of NCM type depended on the dosage. Therefore, the strengthening behavior should be interpreted by considering both factors simultaneously rather than evaluating NCM type or dosage independently.

### 3.2. Fitting Curve Analysis of the Effect of NCM Content on UCS

**Figure 4:**
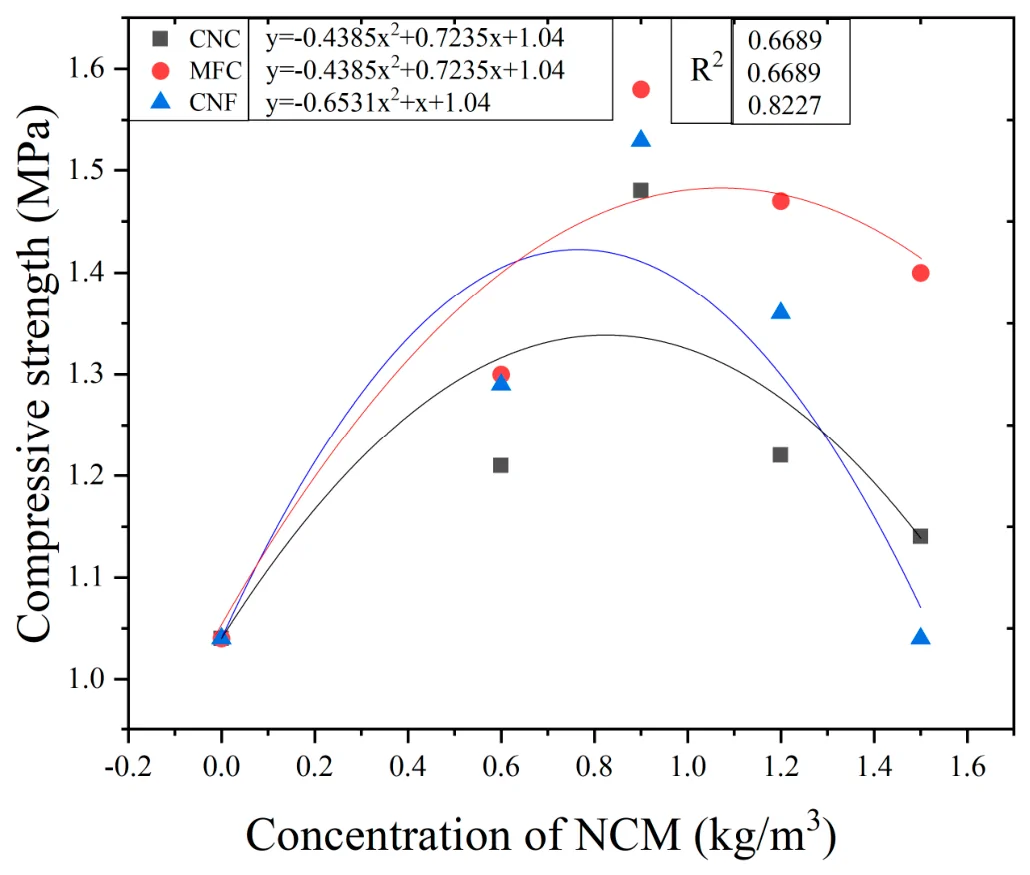
Strength–NCM concentration correlation in CLFTB composites: CNC vs. MFC vs. CNF.

Based on the investigation of nanocellulose content’s regulatory effect on CLFTB mechanical properties, experimental data reveal a significant nonlinear correlation between uniaxial compressive strength (UCS) and nanocellulose concentration, as illustrated in Figure 4. Within the initial concentration range (0–0.6 kg/m^3^), UCS demonstrates a gradient-increasing trend with rising NCM content. Peak mechanical performance is achieved at 0.9 kg/m^3^, where UCS values reach 1.48 MPa (CNC), 1.58 MPa (MFC), and 1.53 MPa (CNF), attributable to nanocellulose’s optimized dispersion mechanism—forming a 3D interpenetrating architecture with the cementitious matrix below the critical concentration threshold, effectively reducing porosity and improving stress transfer efficiency [40], with MFC specimens exhibiting the most favorable structural stability and deformation resistance at this concentration. Concentration-dependent analysis shows that in the low-concentration regime (0–0.6 kg/m^3^), CNF and MFC exhibit nearly identical curve slopes, registering 24% and 25% UCS increases versus control specimens, respectively, indicating comparable reinforcement efficacy. As concentrations progress to the medium-high range (0.6–0.9 kg/m^3^), CNF and CNC curves develop parallel trajectories while MFC attains its strength apex. Within the high-concentration zone (0.9–1.2 kg/m^3^), all formulations initiate a descending trend, characterized by minimal slope reduction for MFC (most gradual attenuation) and maximal slope reduction for CNF (sharpest decline). Finally, in the overdosing regime (1.2–1.5 kg/m^3^), all curves approach experimental minima, with CNF-reinforced CLFTB recording the lowest UCS (1.04 MPa at 1.5 kg/m^3^). Based on the fitted curves in Figure 4, all three nanocellulose types exhibit uniform upward trajectories with stable enhancement rates in the low-concentration regime (0–0.6 kg/m^3^), where CNF and MFC demonstrate comparable enhancement rates—both exceeding CNC’s performance. Beyond the optimal dosage threshold (0.9 kg/m^3^), all specimens manifest strength degradation attributable to stress concentration effects induced by fiber agglomeration, with CNF exhibiting the most pronounced deterioration rate due to its short-fiber characteristics [41]. Specifically for CNF, CLFTB specimens show the steepest UCS ascent within the low-concentration range (0–0.6 kg/m^3^), while beyond the 0.9 kg/m^3^ threshold, further concentration increases trigger significantly more pronounced UCS reductions. Conversely, MFC-enhanced specimens initially yield lower UCS values than CNF at low concentrations (0–0.6 kg/m^3^) yet achieve the highest peak strength (1.58 MPa) at 0.9 kg/m^3^ across the entire experimental program. Notably, MFC maintains superior performance retention with the most gradual strength attenuation during subsequent concentration increases.

**Figure 5:**
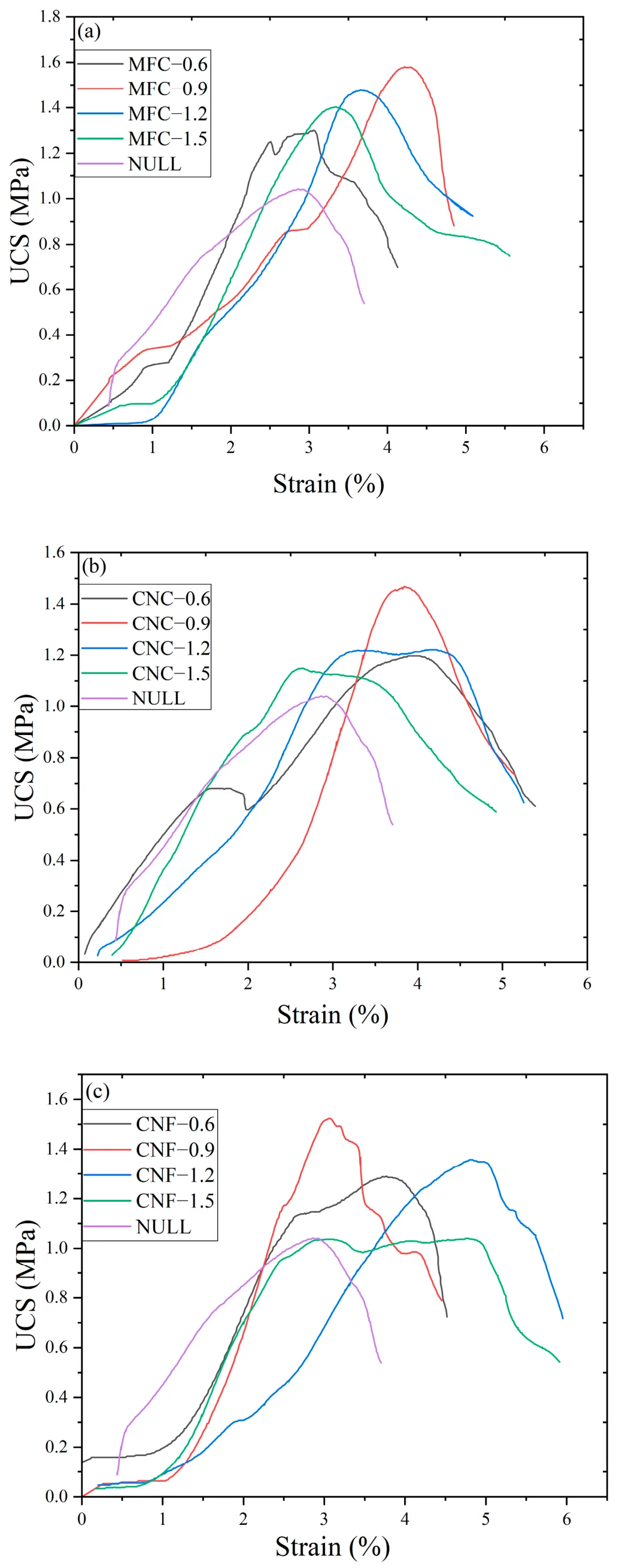
Relationship between UCS and strain curves of CLFTB reinforced with varying types and concentrations of NCM.

The multi-objective optimization analysis from an engineering-application perspective reveals that when the CNF concentration in CLFTB exceeds the threshold (>0.9 kg/m^3^), a sharp strength attenuation occurs (Figure 5), necessitating strict dosage control to avoid strength collapse caused by fiber agglomeration. In contrast, when MFC is added to CLFTB at a concentration of approximately 0.9 kg/m^3^, the compressive strength of CLFTB specimens reaches the highest value among all cellulose types and concentrations, with a relatively mild post-threshold strength decay rate. This broad peak-range characteristic grants MFC superior construction tolerance, making it particularly suitable for mines with significant aggregate variability. CNC exhibits the lowest strengthening efficiency across concentration ranges without a distinct strength peak, yet its dispersion advantage allows it to serve as a supplementary rheology modifier for MFC/CNF systems. Overall, disregarding cellulose costs, MFC demonstrates the most balanced performance enhancement. However, practical cost considerations must be integrated to identify the optimal solution for field implementation.

### 3.3. Failure Modes of CLFTB Specimens

**Figure 6:**
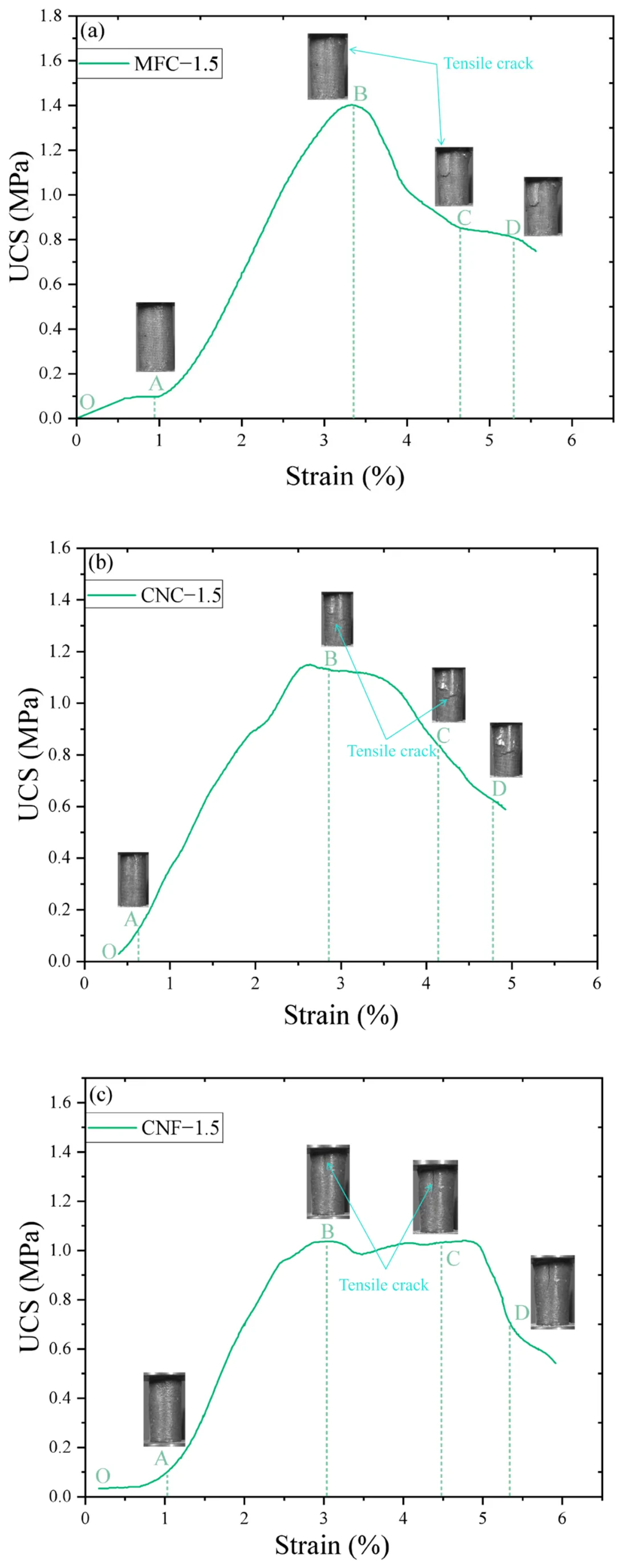
The failure processes of NCM reinforced CLFTB, (**a**) MFC-1.2; (**b**) CNC-1.5; (**c**) CNF-1.5.

As illustrated in Figure 6, the deformation behavior of CLFTB can be categorized into four distinct stages:

(1) Microcrack–Pore Compression Stage: During this phase, internal pore structures within the CLFTB specimen undergo progressive compression under external load, with no visible surface cracks until pores reach a compression-saturated state (Figure 6(a)-OA).
(2) Linear Elastic Stage: Characterized by a linear positive correlation between stress and strain; stress increases proportionally with strain until peak stress is attained, accompanied by minor crack initiation (Figure 6(a)-AB).
(3) Crack Propagation Stage: The linear stress–strain relationship breaks down, with a decreasing curve slope, marking the transition from elastic to plastic deformation. Dominated by internal defects and porosity, the specimen exhibits lateral deformation, crack expansion, and primary crack formation, resulting in a convex-shaped curve (Figure 6(a)-BC).
(4) Crack Coalescence Failure Stage: Stress–strain curves approach failure asymptote, material damage intensifies, and primary cracks fully penetrate the CLFTB specimen, leading to structural collapse (Figure 6(a)-CD).

The failure morphology also varied with the type and dosage of NCM. Compared with the unmodified CLFTB, the incorporation of NCM altered the development and distribution of visible cracks, indicating that the cellulose nanomaterials affected the internal damage evolution of the cemented backfill. The differences were particularly evident near the peak- and post-peak stages, where specimens with different NCM types and dosages exhibited different crack propagation and coalescence characteristics (Figure 6). These morphological differences are consistent with the variations in compressive strength and post-peak stress–strain response observed among the NCM–CLFTB specimens. In general, the modification effect of NCM on failure behavior can be attributed to its influence on the integrity of the cemented matrix and the interaction between the tailings particles and hydration products, thereby affecting the initiation and subsequent propagation of internal cracks.

### 3.4. SEM-EDS Characterization of NCM–CLFTB Composites

**Figure 7:**
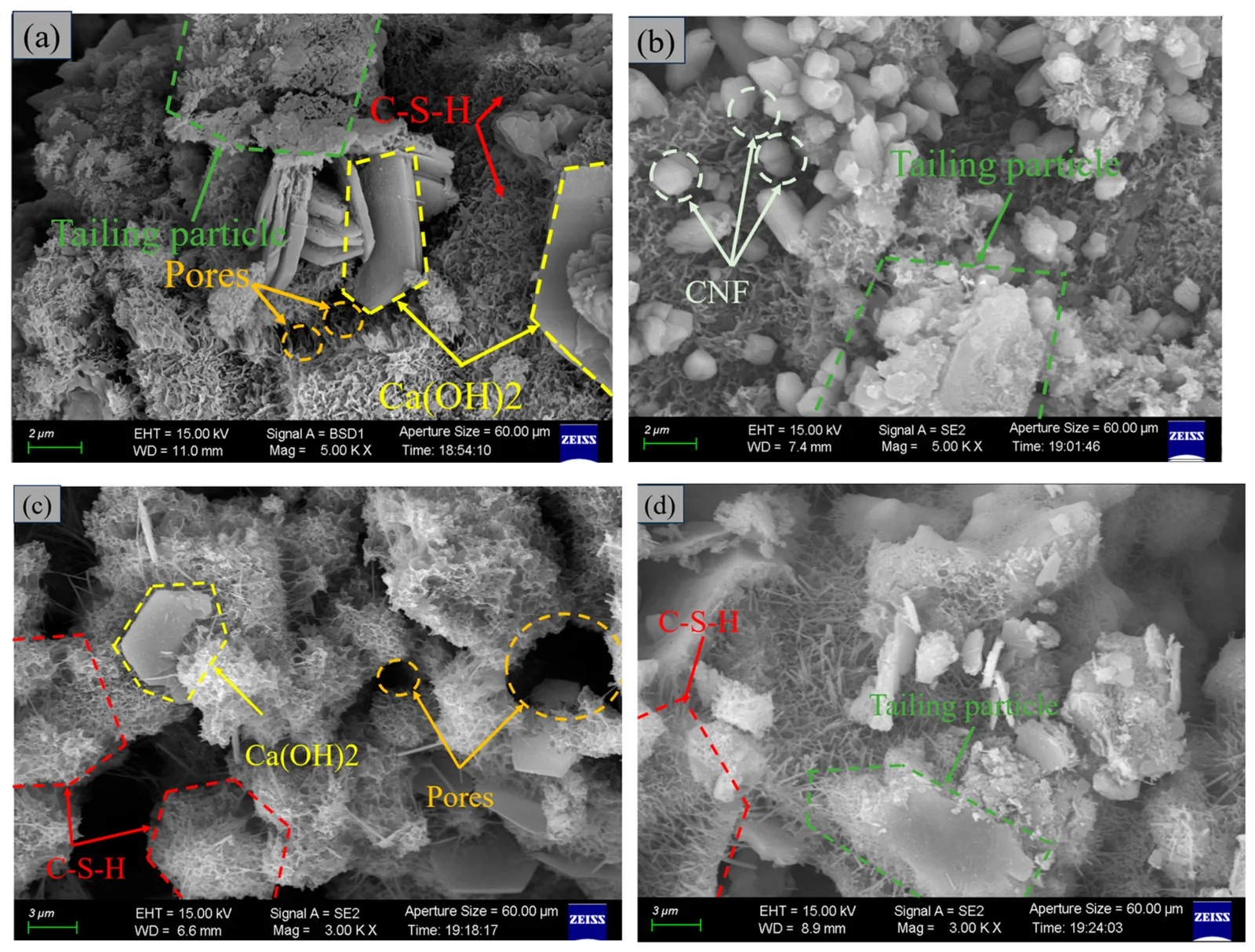
Distribution images of CNF, CNC, and MFC on CLFTB’s fracture surface: (**a**) MFC-0.9; (**b**) CNF-1.2; (**c**) CNC-1.2; (**d**) NULL.

Figure 7 presents the scanning electron microscope (SEM) characterization results of CLFTB incorporating NCM. The hydration products of the cement primarily include flocculent gel (C-S-H), needle-like ettringite (Aft), and some unreacted calcium hydroxide (Ca(OH)_2_), with C-S-H being the main contributor to cement strength. These C-S-H particles thoroughly encapsulate the tailings, and as the curing time increases, both the quantity and distribution of C-S-H gradually increase, improving the pore structure of the filling material and enhancing its strength [42]. Additionally, the needle-like crystal morphology of the secondary hydration product ettringite and the hexagonal plate-like crystals of partially unreacted calcium hydroxide can be identified.

**Figure 8:**
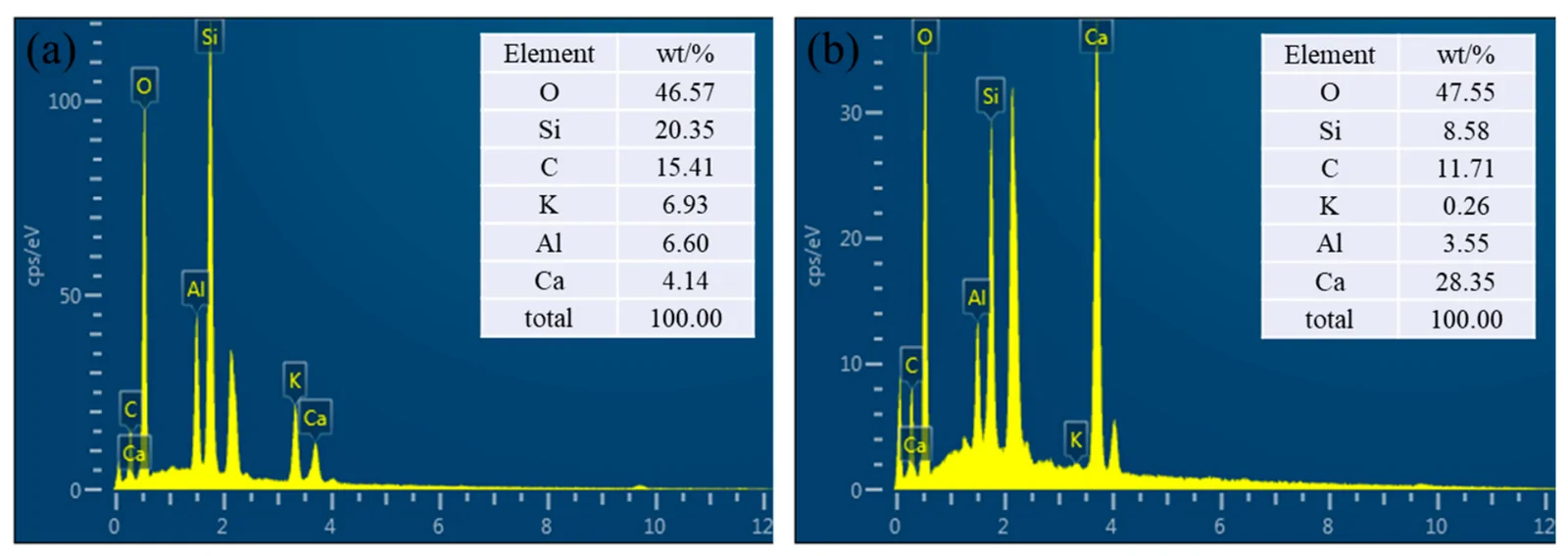
EDS spectra of different regions in CLFTB: (**a**) tailings particle; (**b**) cementitious matrix.

The elemental compositions of different regions in the CLFTB matrix were further analyzed using EDS, as shown in Figure 8. Figure 8a presents the EDS spectrum of a representative tailings particle, whereas Figure 8b corresponds to the cementitious matrix. The tailings particle is mainly enriched in Si, K, and Al, with contents of 46.57, 20.35, and 15.41 wt.%, respectively. In comparison, the cementitious matrix exhibits a higher O content (28.35 wt.%) but substantially lower Al content (0.26 wt.%), together with Si, K, and Ca contents of 47.55, 11.71, and 3.55 wt.%, respectively. These differences indicate the distinct local chemical compositions of the tailings particle and cementitious matrix.

The present SEM-EDS characterization mainly provides information on the morphology and elemental distribution of the NCM–CLFTB composites. Although these observations provide useful evidence for interpreting the microstructural changes associated with NCM incorporation, SEM-EDS alone cannot directly verify the formation of specific hydration products, chemical bonds, or phase transformations. Further characterization using XRD, TGA, and FTIR would help clarify phase evolution, thermal characteristics, and chemical interactions between NCM and the cementitious matrix. These analyses will be considered in future investigations.

## 4. Conclusions

The experimental analysis above demonstrates that incorporating nanocellulose significantly enhances the mechanical properties of CLFTB, with a compressive strength improvement of 28.6%. This approach not only achieves efficient resource utilization of lithium mica beneficiation tailings but also substantially reduces raw material costs by partially replacing traditional cementitious components. Through systematic preparation of 39 experimental specimens with varying formulations, this study characterized the macroscopic mechanical evolution via unconfined compressive strength (UCS) testing and elucidated the regulatory mechanisms of nanocellulose on hydration product morphology and elemental distribution using SEM-EDS analysis. Key conclusions are as follows:

1. Experimental results confirm that nanocellulose incorporation markedly enhances mechanical performance. Compressive strength initially increases and then decreases nonlinearly with ascending nanocellulose dosage. Notably, microfibrillated cellulose (MFC)-modified CLFTB exhibits the optimal reinforcement: at 0.9 kg/m^3^ dosage, its compressive strength reaches 1.58 MPa, representing a 51.9% increase over the control group (1.04 MPa), with statistically significant differences.
2. Post-peak strength degradation rates are lowest in MFC-modified CLFTB, as evidenced by smoother critical-strength decline curves compared to CNC- and CNF-modified systems, indicating superior stability for practical applications.
3. Mechanical response analysis reveals a four-stage failure evolution in CLFTB: initial pore compaction stage; microcrack closure and structural densification; linear elastic deformation phase; stress–strain behavior conforms to Hooke’s law; steady-state crack propagation stage; progressive damage accumulation; final instability stage; macro-crack network coalescence as the failure marker.

## Figures and Tables

**Table 1:** The oxide composition of lithium-feldspar tailings.

Oxide Name	SiO_2_	Al_2_O_3_	CaO	Fe_2_O_3_	MgO	SO_3_	Li_2_O	LOI	Others
Content (%)	39.8	15.3	16.9	3.98	1.40	9.74	0.26	3.53	9.09

**Table 2:** Key characterization parameters for various forms of nanocellulose-based materials.

Category	Model	Preparation Method	State	Surface Group	Diameter	Length
Carboxylated Cellulose Nanofibers	CCNF	Carboxylated	99.6% powder	-COOH	3–20 nm	Greater than 1 μm
Cellulose Nanocrystals	CNC	Hydroxylation	99.6% powder	-OH	3–20 nm	100–300 nm
Microfibrillated Cellulose	MFC	Mechanical method	Aqueous dispersion	-OH	0.1–1 μm	Greater than 20 μm

**Table 3:** Proportional blending of CLFTB with varying nanocellulose dosages.

Sample	Cement (g)	Tailings (g)	Total Mass of Cement and Tailings (g)	Water (g)	Cement-to-Tailings Ratio	Nano Cellulose (g)
S1	100	600	700	233.3	1:6	0
S2	100	600	700	233.3	1:6	0.1399
S3	100	600	700	233.3	1:6	0.2099
S4	100	600	700	233.3	1:6	0.2799
S5	100	600	700	233.3	1:6	0.3499

**Table 4:** Two-way ANOVA results for the UCS of NCM-modified cemented lithium-tailings backfill.

Source	Sum of Squares	df	F	*p*-Value
NCM type	0.19995	2	248.437	<0.001
NCM dosage	0.56690	3	469.580	<0.001
NCM type × dosage	0.13105	6	54.276	<0.001
Error	0.00966	24	—	—

## Data Availability

The original contributions presented in this study are included in the article. Further inquiries can be directed to the corresponding author.
